# A primary healthcare approach to children with fever

**DOI:** 10.4102/safp.v68i1.6199

**Published:** 2026-02-23

**Authors:** Indiran Govender, Henry I. Okonta

**Affiliations:** 1 Department of Family Medicine and Primary Healthcare, Walter Sisulu University, Mthatha, South Africa; 2 Department of Family Medicine and Primary Healthcare, Faculty of Health Sciences, Sefako Makgatho Health Sciences University, Pretoria, South Africa

**Keywords:** antibiotics, children, fever, infections, National Institute for Health and Care Excellence Guidelines, antipyretic

## Abstract

A child with fever presenting with concerned caregivers is a common occurrence in primary healthcare in South Africa. Many fevers are self-limiting. However, serious and possibly fatal diseases must be excluded before discharging the child. This article is a guideline for primary care practitioners, which needs to be read in conjunction with the management guidelines for specific diseases.

## Introduction

Fever is a physiological response to disease, which facilitates and accelerates recovery. Fever is a frequent complaint at presentation, although there is no evidence that children with fever are at increased risk for serious outcomes. Parents are worried about risks such as seizures, brain damage, or death.^1,2^ Caregivers are also not confident about how to manage fever in children, as there are many opinions from family members. Many drugs can be bought over the counter or prescribed to bring down fevers quickly in a sick child.^2^ The most frequently used over-the-counter (OTC) medicines are paracetamol and ibuprofen.^1,2^

However, although a common presenting complaint, fever is mostly self-limiting and is usually a result of a self-limiting viral infection. Sometimes, fevers may be caused by serious bacterial infections, such as meningitis and pneumonia, and other non-infectious illnesses. Thus, the underlying illness causing the fever needs to be determined, and it is important to be able to distinguish between a child with a high risk of a serious illness who requires specific treatment, hospitalisation, or specialist care, and those at low risk who can be managed safely at home. This article will assist primary healthcare workers and general practitioners in identifying high-risk children who present with fever, and deciding on the correct time to refer, the appropriate use of antipyretic medication, and the correct advice to be given to parents and caregivers.

## Definition

There is no universal agreement as to the upper limit of the normal temperature range in humans.^3^ Body temperatures vary according to the location at which measurements are taken (oral, rectal, tympanic, or skin thermal scan); the type of thermometer used (contact, noncontact, or handheld thermal scanner); the time of the day; age of the patient, sex, and race.^3^ The World Health Organization^4^ defined fever as an axillary temperature of > 37.5°C, while other authors defined fever as a body temperature of > 38°C.^5,6,7,8^ Fever without a focus is an acute febrile illness, typically less than 14 days duration, with no identifiable cause after careful history and physical examination. Chronic fever is a fever that lasts for more than 14 days, and fever of unknown origin refers to a daily or intermittent fever of at least 14 days with an uncertain cause after careful history, physical examination, and initial laboratory assessment.^5,9^

## Aetiology

Fever in children is caused by infectious and non-infectious conditions, with non-infectious disorders further categorised as inflammatory, malignant, and miscellaneous. The important infectious causes of fever that must be considered are listed in Table 1.^9^ In the South African context, given that infections are one of the quadruple burden of diseases, it is important to rule out common infections such as human immunodeficiency virus (HIV), malaria, and tuberculosis (TB).^6,9^ The South African Primary Care guidelines are a good reference for the various causes of fever, including infections; however, this information is discussed under headings such as HIV, skin conditions, TB, among others. rather than a general topic of fever.^10^ The inflammatory disorders include mainly autoimmune conditions such as Kawasaki disease, juvenile idiopathic arthritis, inflammatory bowel diseases, and systemic lupus erythematosus. Leukaemia, lymphoma, and neuroblastoma are some of the childhood malignancies that can cause fever. The miscellaneous category of causes of fever includes drugs, toxins, post-immunisation, familial dysautonomia, and factitious disorder.

**TABLE 1:** Common infectious causes of fever in children.

Neonatal or infant infections up to 3 months of age	Infections beyond 3 months of age
Congenital infections Toxoplasmosis Rubella Herpes Simplex Varicella-Zoster *Erythema infectiosum* Cytomegalovirus HIV Hepatitis B or Hepatitis C Group B Streptococci *Escherichia coli* and other enteric pathogens *Listeria monocytogenes* Non-congenital infections Respiratory syncytial virus Influenza Parainfluenza Adenovirus Rhinovirus Corona viruses Cytomegalovirus Epstein-Barr virus Human herpes virus 6 *Salmonella* *Staphylococcus aureus* *Streptococcus pneumoniae* Malaria	Bacteraemia, sepsis, and meningitis caused by S. *pneumoniae* and *Neisseria meningitidis* Encephalitis Otitis media and pneumonia caused by *S. pneumoniae, Haemophilus influenzae*, and *Moraxella catarrhalis* Urinary tract infection caused by *E. coli* and other enteric pathogens Enteritis caused by *Salmonella* and *Shigella* Skin and soft tissue infections caused by *S. aureus* and Group A Streptococci Bone and joint infections caused by *S. aureus, Salmonella*. and *Kingella kingae* Pharyngitis and Scarlet fever caused by Group A Streptococci Sinusitis Tuberculosis Rickettsial infections Malaria Measles Typhoid HIV and/or AIDS Influenza

*Source*: Adapted from Consolini DM. Fever in infants and children [homepage on the Internet]. 2025 [cited 2025 Jul 08]. Available from: https://www.msdmanuals.com/home/children-s-health-issues/symptoms-in-infants-and-children/fever-in-infants-and-children^9^

HIV, human immunodeficiency virus; AIDS, acquired immunodeficiency syndrome.

The causes of febrile illnesses differ based on whether the fever is acute, acute recurrent or periodic, or chronic.

Most acute fevers (fevers of less than 14 days’ duration) in children are caused by infection with viral respiratory or gastrointestinal infections and certain bacterial infections (otitis media, pneumonia, and urinary tract infections). Potential infectious causes of acute fever also vary with the age of the child. Neonates are considered functionally immunocompromised and at higher risk of serious infections caused by organisms acquired during the perinatal period, such as group B streptococci, *Escherichia coli*, and *Listeria monocytogenes*, which can cause bacteraemia, and herpes simplex, which can cause viraemia.^9^

Most acutely febrile children in the age group of 1 month to 2 years have self-limiting viral diseases. A minority of these children may be in the early stage of a serious infection, and the main concern in such children is whether occult bacteraemia is present. *Streptococcus pneumoniae* and *Haemophilus influenzae* type b are the most common causes of occult bacteraemia.^9^

The non-infectious causes of acute fevers include Kawasaki disease, acute rheumatic fever, diabetes insipidus, anhidrosis, heatstroke, dysautonomia, toxic ingestion of medications with anticholinergic effects, measles vaccination, and pertussis vaccination. Fever with a history of travel to disease-endemic areas should prompt the suspicion of travel-related infections (Table 2).

**TABLE 2:** Some travel-related infections.

Food-borne- and/or water-borne diseases Traveller’s diarrhoea Cholera Typhoid Hepatitis A Hepatitis E Contaminated soil and/or water-borne diseases Cutaneous larva migrans Schistosomiasis Leptospirosis Mosquito-borne diseases Malaria Dengue fever Yellow fever Zika

The causes of chronic fever can be infectious and non-infectious. Some of the common causes of chronic fever in children are listed in Table 3.

**TABLE 3:** Some causes of chronic fever in children worldwide.

More common infectious causes: Tuberculosis Malaria Abscesses, which can be intra-abdominal, hepatic, or renal Osteomyelitis and septic arthritis Sinusitis HIV Less common infectious causes: Infective endocarditis Infectious mononucleosis Cat-scratch disease Histoplasmosis	More common non-infectious causes: Systemic rheumatic diseases such as Juvenile idiopathic arthritis, Systemic lupus erythematosus, and Acute rheumatic fever Less common non-infectious causes: Malignancies such as lymphoma, leukaemia, neuroblastoma, and sarcoma Inflammatory bowel disease Medications Factitious fever

*Source*: Adapted from Consolini DM. Fever in infants and children [homepage on the Internet]. 2025 [cited 2025 Jul 08]. Available from: https://www.msdmanuals.com/home/children-s-health-issues/symptoms-in-infants-and-children/fever-in-infants-and-children^9^

HIV, human immunodeficiency virus.

## Pathophysiology

**FIGURE 1:**
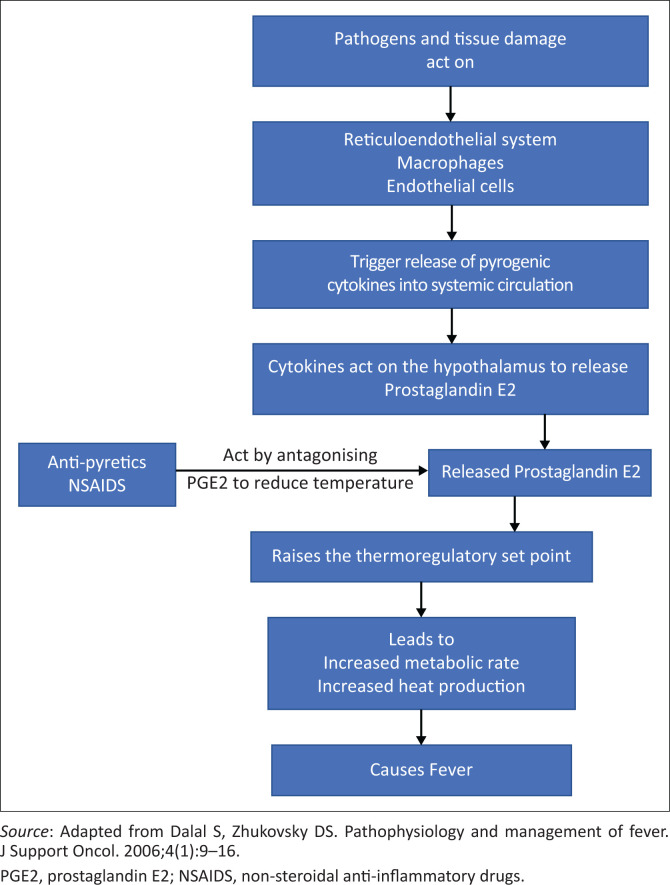
The pathophysiologic pathway of fever.

The body’s temperature is tightly regulated between 36 °C and 37.8 °C, mediated, and controlled by the central nervous system in the pre-optic area of the hypothalamus. Pathogens and tissue damage result in the release of cytokines by the reticuloendothelial system, macrophages, and endothelium. These cytokines reach the hypothalamus through the systemic circulation and trigger≈the release of prostaglandin E2 (PGE2). Prostaglandin E2 raises the thermoregulatory set point to a higher temperature, which leads to increased metabolic rate.^11^ The increased metabolic rate produces heat, which results in fever (Figure 1).

Non-steroidal anti-inflammatory drugs (NSAID) such as ibuprofen and other antipyretics such as paracetamol lower temperature by antagonising PGE2 release. It should be noted that non-PGE2-mediated processes can cause fever as well, including increased oxygen consumption, metabolic demands, protein breakdown, and gluconeogenesis. In contrast to fever (which rarely goes above 41 °C), which is induced by cytokine activation and leads to thermoregulatory responses, hyperthermia refers to elevation of core body temperature above ‘set point’ as a result of the failure of body thermoregulation and is usually a result of excessive exogenous or endogenous heat production – heat exhaustion, heat cramps, heat stress, and heatstroke.^1,6,12^

As a rule, for every 1°C core body temperature increase, the heart rate and/or pulse increases by 10 beats/min – 20 beats/min, and the respiratory rate increases by 2 breaths/min – 4 breaths/min (Liebermeister’s rule).^13,14^ While tachycardia is often a normal physiologic response to increased metabolic rate caused by fever, some febrile illnesses may manifest with an unusual association of fever with relative bradycardia. This phenomenon is referred to as sphygmothermic dissociation or Faget sign, and occurs in diseases such as typhoid fever, yellow fever, dengue fever, and brucellosis.^15^

## Evaluation of children with fever

Fever is a symptom of an underlying illness that needs to be identified with a thorough medical history, complete physical examination, and appropriate tests or investigations if indicated.

### History

The history of the presenting complaint should elicit the onset, duration, and pattern of the fever, as well as any associated symptoms. The associated symptoms may suggest the serious nature of the febrile illness or indicate the underlying cause of the fever. The history should note any predisposing factors to infection. These predisposing factors include recent exposures to infection in a family member, caregiver, school, or crèche, as well as indwelling medical devices. In neonates, additional predisposing factors include prematurity, prolonged rupture of membranes, and positive prenatal tests for group B streptococcus, cytomegalovirus, or sexually transmitted infections.

The past medical history should cover previous febrile illnesses and any known conditions that predispose to infections, such as recent surgery, congenital heart disease, sickle cell anaemia, cancer, and HIV, and/or acquired immunodeficiency syndrome (AIDS). The medication history should enquire about any medicines or other remedies administered for the fever, and the child’s response. It should also elicit any current and recent use of antibiotics and any recent vaccinations. The immunisation status of the child should be elicited and recorded.

Recent travel history, especially to malaria-endemic areas, and environmental exposures to mosquitoes, ticks, cats, farm animals, and reptiles should be noted.

### Physical examination

A complete physical examination is mandatory in all children presenting with fever. The complete head-to-toe examination seeks to identify any focus of infection and signs that predict the risk of serious illness. The traffic light system is a useful tool for identifying children at risk of serious illness (Table 4). In this system, children with fever and any of the signs or symptoms in the red column are at high risk of serious illness; children with fever and any of the signs or symptoms in the amber column and none in the red column are at intermediate risk; and children with signs and symptoms in the green column and none in the amber or red columns are at low risk.^16^ This is one example of a triage tool, and the purpose of all of these tools is to identify and rapidly intervene for those who need urgent medical intervention. The National Institute for Health and Care Excellence Guidelines (NICE) traffic light system is a good choice in this setting.

**TABLE 4:** Traffic light system for identifying risk of serious illness.

Examination of child	Green: Low risk	Amber: Intermediate risk	Red: High risk
Colour	Normal colour	Pallor reported by parent or carer	Pale, mottled, ashen or blue
Activity	Responds normally to social cues Content or smiles Stays awake or awakens quickly Strong normal cry or not crying	Not responding normally to social cues No smile Wakes only with prolonged stimulation Decreased activity	No response to social cues Appears ill to a healthcare professional Does not wake or if roused does not stay awake Weak, high-pitched, or continuous cry
Respiratory		Nasal flaring Tachypnoea: respiratory rate > 50 breaths per minute, age 6 months to 12 months. > 40 breaths per minute, age more than 12 months old Oxygen saturation less than or equal to 95% in air Crackles in the chest	Grunting Tachypnoea: respiratory rate more than 60 breaths per minute Moderate or severe chest indrawing
Circulation and hydration	Normal skin and eyes Moist mucous membranes	Tachycardia: More than 160 beats per minute, age less than 12 months old More than 150 beats per minute, age 12 months to 24 months More than 140 beats per minute, age 2 years old to 5 years old Capillary refill time more than or equal to 3 sec Dry mucous membranes Poor feeding in infants Reduced urine output	Reduced skin turgor
Other	None of the amber or red symptoms or signs	Ages 3 months to 6 months, temperature more than or equal to 39°C Fever for more than or equal to 5 days Rigors Swelling of a limb or joint Non-weight-bearing limb or not using an extremity	Age less than 3 months, temperature more than or equal to 38°C Non-blanching rash Bulging fontanelle Neck stiffness Status epilepticus Focal neurological signs Focal seizures

*Source*: NICE guidline 143. Fever in under 5 s: Assessment and initial management [homepage on the Internet]. 2019. Available from: http://www.nice.org.uk/guidance/ng143^16^

### Investigations

The investigations in children with fever are directed by findings in the history, on physical examination, and on clinical suspicion. The investigations required for the evaluation of all neonates with acute fever include WBC (white blood cell) count with differential, blood cultures, urinalysis and urine culture, lumbar puncture (for cerebrospinal fluid analysis and culture), and the inflammatory markers C-reactive protein and procalcitonin.^8^ Chest x-ray is required for neonates with respiratory manifestations, and stool culture is performed for neonates with diarrhoea.^8^ Routine screening for malaria should be performed in all febrile children (including neonates) from a malaria-endemic area.^1,17^ It can be carried out by Giemsa staining under microscopy and rapid diagnostic tests for antigen detection.

The same set of investigations is required in infants 1-month-old to 3 months old with acute fever, who are assessed as intermediate or high risk for serious illness based on their history and physical examination findings.^8^ In acutely febrile infants from 1 to 3 months old, who are assessed as low risk for serious illness, the lumbar puncture is deferred, pending the outcome of the initial investigations.^9,18^

Children with acute fever in the 3 months to 36 months age bracket with intermediate or high-risk assessment and those with low-risk assessment but not fully immunised require evaluation for serious bacterial infection with WBC count and differential, blood culture, urinalysis and urine culture, and chest X-ray if they are tachypnoeic or have a WBC count of < 20 x 10^9^/L. In children above 36 months old, the required investigations are directed by the history and physical examination findings.^9^

In children with chronic fever, the set of investigations for the initial evaluation includes full blood count (FBC) with differential, erythrocyte sedimentation rate and C-reactive protein, blood cultures, urinalysis and urine culture, chest X-ray, serum urea, creatinine, electrolytes, albumin, liver enzymes, HIV serology, and tuberculin test.^9^ The results of these tests may indicate the need for further investigations.

### Management of fever in children

The treatment of fever is directed at the underlying cause if known. Risk-stratification strategies such as the Rochester, Boston, and Philadelphia criteria are used to assess the risk of occult bacteraemia in febrile children.^8^ Infants who meet these criteria are considered low risk for serious bacterial infection (SBI) and can be managed as outpatients. The infants who are not considered low risk should be admitted or referred to appropriate facilities.

The Rochester criteria are used to identify infants aged 28–60 days, who have a low risk of occult bacteraemia and include the following^19^:

- Well-appearing infant.
- Born at term > 37 weeks, at home with or before mother, no prior hospitalisations, no prior antibiotic use, no prior treatment for unexplained hyperbilirubinemia, and no chronic disease.
- The infant has no evidence of skin, soft tissue, bone, joint, or ear infection.
- The infant has the following laboratory values: WBC count 5–15 × 10^9^/L, absolute band count of less than 1.5 × 10^9^/L, urinalysis with less than 10 WBCs/HPF (high-power field), and stool with less than 5 WBC/HPF if the infant has diarrhoea.

The Boston criteria, developed to identify febrile infants aged 28–89 days, who have a low risk of occult bacteraemia, include the following^20^:

- No immunisations or antimicrobials within the preceding 48 h.
- No evidence of dehydration or ear, soft tissue, or bone infection.
- Well-appearing.
- The caretaker is available by telephone.
- The infant has the following laboratory values: WBC count less than 20 × 10^9^/L, CSF with less than 10 cells/µL, urinalysis with less than 10 WBCs/HPF, and no infiltrate on chest x-ray if done.

The Philadelphia criteria, used to identify febrile infants aged 29–60 days with low risk of occult bacteraemia, include the following^8^:

- Well-appearing.
- WBC count less than 15 × 10^9^/L.
- Band-neutrophil ratio less than 0.2.
- Urinalysis with less than 10 WBC/HPF and a negative Gram stain result.
- CSF has less than 8 WBCs/µL and a negative CSF Gram stain.
- Chest X-ray with no infiltrate if done.
- Stool has no blood and few or no WBCs on the smear.

**FIGURE 2:**
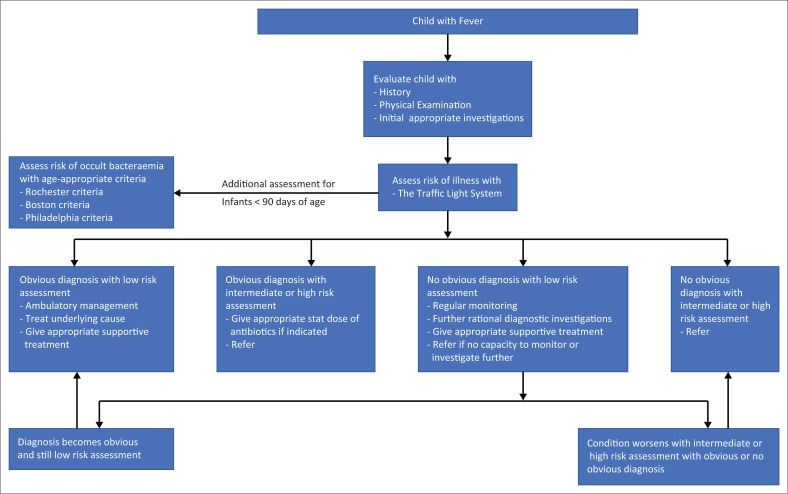
An approach to the evaluation of a child with fever.

An approach to the evaluation of a child with fever, incorporating appropriate risk-assessment tools (the traffic light system, Rochester criteria, Boston criteria, and Philadelphia criteria), is illustrated in Figure 2.

Febrile neonates are considered high risk, based on age alone, and should be admitted for in-hospital treatment.^18^ Infants 1–3 months old with fever without a focus, who are assessed as low risk of SBI, should be monitored and re-evaluated within 24 h to 48 h or sooner if the condition deteriorates. Infants 1–3 months old with fever without a focus, who have a high risk of SBI, should be admitted to the hospital and started on empiric parenteral antibiotics (after obtaining appropriate culture specimens), pending the outcome of culture results.^21^ The empiric antibiotic therapy should be comprehensive to cover likely pathogens in the clinical setting. Ampicillin in combination with gentamycin,^22^ ampicillin in combination with cefotaxime,^22,23^ or cefotaxime alone^23^ are the recommended empiric therapies in neonates. In infants in the 1–3 months age group, ceftriaxone or cefotaxime are the appropriate antibiotics.^22^ Empiric antibiotic treatment is not needed for infants and children 3–36 months old, who have normal urinalysis and no localising signs.^22^ In selecting the most appropriate antibiotic for children, it is important to consider the South African context and refer to the Primary Health Care (PHC) Essential Medicines List and Integrated management of childhood illness (IMCI) guidelines for antibiotic choices.^4,24^

Antipyretics are used to make the child more comfortable and are not used primarily for the reduction of temperature. They do not reduce the risk of febrile convulsions.^25,26^ Discomfort during a febrile illness is often a result of associated pain from myalgia, headache, sore throat, etc.^6^ Paracetamol and ibuprofen are safe and effective for short-term use in children and are the drugs of choice to manage fever.^27,28^ Dosing of antipyretics should be accurately based on body weight and not just estimated.^1^ For children older than 3 months, the dose of paracetamol is 15 mg/kg every 6 h as necessary, and the dose of ibuprofen is 10 mg/kg every 6 h as necessary.^27^ In children who are unable to consume orally, suppositories may be used, but the absorption and bioavailability are more variable than the oral route is.^29^ IV paracetamol is an equally effective alternative in children who cannot ingest orally.^30^ Tepid sponging does not alleviate discomfort in febrile children and is therefore discouraged.^10,31,32^

Adequate fluid intake is necessary to prevent dehydration, which can be exacerbated by fever. The intake should replace any fluid losses (from vomiting and/or diarrhoea) and cover the maintenance fluid needs, which are higher in febrile children.^5^

## Conclusion

Fever is one of the leading causes of presentations to PHC facilities in children and signifies a normal body response to an underlying inflammatory disorder. The anxiety and stress in the caregiver also need to be managed empathetically. Fever is mostly caused by infectious agents, and the focus of the healthcare practitioner is to systematically identify and manage the offending infection, being mindful of the likely causative agent in the child’s age group. While most children with uncomplicated fever can be managed on an outpatient basis in the PHC, those who present with danger signs should be stabilised and referred for hospital-based management.

In this article, we sought to provide information about the commonest causes of fever in children and to provide the primary healthcare doctor with an approach to managing this common presentation. This information will also help to allay the fears of caregivers. A limitation of this article is that the focus is on the South African context, and a general approach to fever in children is discussed; it does not go into detail on any specific condition.
